# Functional Genetic Polymorphisms in PP2A Subunit Genes Confer Increased Risks of Lung Cancer in Southern and Eastern Chinese

**DOI:** 10.1371/journal.pone.0077285

**Published:** 2013-10-29

**Authors:** Rongrong Yang, Lei Yang, Fuman Qiu, Lisha Zhang, Hui Wang, Xiaorong Yang, Jieqiong Deng, Wenxiang Fang, Yifeng Zhou, Jiachun Lu

**Affiliations:** 1 The Institute for Chemical Carcinogenesis, The State Key Lab of Respiratory Disease, Guangzhou Medical University, Guangzhou, China; 2 Soochow University Laboratory of Cancer Molecular Genetics, Medical College of Soochow University, Suzhou, China; Universitat Pompeu Fabra, Spain

## Abstract

Protein phosphatase-2A (PP2A) is one of the major cellular serine-threonine phosphatases and functions as a tumor suppressor that negatively regulates the activity of some oncogenic kinases. Recent studies have reported that PP2A expression was suppressed during lung carcinogenesis, we there hypothesized that the single nucleotide polymorphisms (SNPs) in PP2A subunit genes may affect PP2A function and thus contribute to lung cancer susceptibility. In a two-stage case-control study with a total of 1559 lung cancer patients and 1679 controls, we genotyped eight putative functional SNPs and one identified functional SNP (i.e., rs11453459) in seven major PP2A subunits (i.e., *PPP2R1A*, *PPP2R1B*, *PPP2CA*, *PPP2R2A*, *PPP2R2B*, *PPP2R5C*, *PPP2R5E*) in southern and eastern Chinese. We found that rs11453459G (-G/GG) variant genotypes of *PPP2R1A* and the rs1255722AA variant genotype of *PPP2R5E* conferred increased risks of lung cancer (rs11453459, -G/GG vs. –: OR = 1.31, 95% CI = 1.13–1.51; rs1255722, AA vs. AG/GG: OR = 1.27, 95% CI = 1.07–1.51). After combined the two variants, the number of the adverse genotypes was positively associated with lung cancer risk in a dose-response manner (*P*_trend_  = 5.63×10^−6^). Further functional assay showed that lung cancer tissues carrying rs1255722AA variant genotype had a significantly lower mRNA level of PPP2R5E compared with tissues carrying GG/GA genotypes. However, such effect was not observed for the other SNPs and other combinations. Our findings suggested that the two functional variants in *PPP2R1A* and *PPP2R5E* and their combination are associated with lung cancer risk in Chinese, which may be valuable biomarkers to predict risk of lung cancer.

## Introduction

Reversible phosphorylation of proteins is an important regulatory mechanism for maintaining cell homeostasis that regulates cell growth, proliferation, apoptosis, survival and differentiation [1]. It balances phosphorylation-dependent signal transduction pathways by virtue of the phosphorylation with protein kinases and dephosphorylation with protein phosphatases. Multiple evidences have indicated that the aberrant activity of phosphorylation involves the development of several cancers (e.g., lung cancer), which was caused by activated oncogenic kinases and inactivated phosphatases [2]. Inactivated phosphatases would lead to aberrant activation of oncogenic signaling pathways, and ultimately cause tumorigenesis [3], [4]. Dysfunctional phosphatases have been observed in various tumors with genetic or functional alterations [2].

The serine-threonine protein phosphatase 2A (PP2A) is one of the major cellular Ser/Thr protein phosphatases which plays key roles in regulating cell growth [5], apoptosis [6], transformation [7] and causes dephosphorylation in several signaling pathways such as MAP kinase signaling and WNT signaling [8], [9]. Multiple evidences have suggested that PP2A functions as a tumor suppressor [10], [11] by inhibiting several oncogenic kinases_ENREF_11 such as c-Myc and AKT [12]–[14], and tumor suppressors like p53 [15]. In contrast, inactivated PP2A would promote tumorgenesis by advancing cell proliferation and survival [16]_ENREF_19_ENREF_20. Dysfunctional PP2A has been observed in various human cancers including lung cancer, which may be due to genetic or epigenetic changes in different PP2A subunit genes [17], [18].

The PP2A is a trimeric holoenzyme consisted of a scaffolding A subunit, one regulatory B subunit and a catalytic C subunit [19]. Typically, the structural core subunit PP2Aa (PPP2R1A/PPP2R1B) interacted with the catalytic subunit PP2Ac (PPP2CA/PPP2CB) to make up the core of the enzyme, and the binding of the widely varied B regulatory subunits (15 genes) to the core enzyme results in tissue-expressed specificity and substrate specificity of the PP2A holoenzyme complexes. Recently, several studies have reported that genetic variants in these PP2A subunit genes were associated with various human diseases including cancer [20]–[22]. Remarkably, the results from one genome-wide association study (GWAS) conducted in Chinese, in which we previously participated, identified a intron single nucleotide polymorphism (SNP) near one B regulatory subunit (*PPP2R2B*) to be a lung cancer susceptible locus, reflecting an important role of in PP2A on lung cancer susceptibility [23]. However, no study has yet systematically tested the associations between genetic variants in PP2A subunit genes and lung cancer risk. Therefore, in current study, we tested the hypothesis that the genetic variants in PP2A subunit genes may alter the susceptibility of lung cancer.

Because the PP2A has tissue-expressed specificity, we selected genetic variants of these PP2A subunits with function in lung based on previous published articles (i.e., *PPP2R1A* [24], *PPP2R1B* [25], *PPP2CA* [26], *PPP2R2A* [27], *PPP2R2B* [23], *PPP2R5C* [28] and *PPP2R5E* [29]). In a two-stage case-control study, we genotyped nine putative functional SNPs of above genes in southern Chinese and validated the promising SNPs in eastern Chinese to analyze the associations between them and lung cancer risk. The effect of promising SNPs on gene expression was further detected.

## Materials and Methods

### Study subjects

In this study, two independent case-control samplings including a southern Chinese population as a discovery set and an eastern Chinese population as a validation set were used as previously described [30]–[34]. In brief, there were 1056 histopathologically confirmed primary lung cancer cases and 1056 age (±5 years) and sex frequency-matched cancer-free controls in the discovery set, and 503 newly diagnosed lung cancer patients and 623 age (±5 years) and sex frequency-matched healthy controls in the validation set. All the participants were genetically-unrelated ethnic Han Chinese and none had blood transfusion in the last 6 months. Having given a written informed consent, each participant was scheduled for an interview with a structured questionnaire to collect selected information, and to donate 5ml peripheral blood. The definition of smoking status, pack-years smoked, drink status and family history of cancer have been described previously [32], [35]. The study was approved by the institutional review boards of Guangzhou Medical University and Soochow University.

### SNP selection

By searching the dbSNP database (http://www.ncbi.nlm.nih.gov/), we found there were nine putative functional SNPs in the aforementioned seven genes which are located in the predicted 2000 bp promoter, coding region and 3′-untranslated region (3′-UTR) with minor allele frequency (MAF) >5% in Han Chinese. They are rs13344984T>C in promoter, rs10421191G>A in 3′-UTR of *PPP2R1A*, rs2850247 C>A and rs612345 A>G in promoter of *PPP2R1B*, rs7840855C>T in promoter of *PPP2R2A*, rs3742424G>C in coding region of *PPP2R5C* (causing an amino acid change from Alanine to Proline at codon 476), rs1255720T >C and rs1255722G >A in promoter of *PPP2R5E,* rs2292283G >A in promoter of *PPP2CA*. The linkage disequilibrium (LD) analysis further showed that the two SNPs (rs1255720T >C and rs1255722G >A) of *PPP2R5E* were in completely LD with each other (r^2^ = 0.309, D' = 1.0), we therefore selected one of them (rs1255722G >A) in current study. Furthermore, Yu-Chun Lin et.al have identified a SNP rs11453459->G within the promoter of *PPP2R1A* is functional and common with MAF >5% in Chinese, we also selected this SNP albeit it was not reported in dbSNP with frequency data of CHB [36]. However, no such SNP was observed for *PPP2R2B*. Taken together, we selected nine SNPs of PP2A subunit genes (rs10421191G >A, rs11453459-/G and rs13344984T >C of *PPP2R1A*, rs2850247 C >A and rs612345 A >G of *PPP2R1B*, rs7840855C >T of PPP2R2A, rs1255722A >G of *PPP2R5E*, rs3742424G >C of *PPP2R5C*, rs2292283A >G of *PPP2CA*) in current study.

### Genotype analysis

The Taqman allelic discrimination assay was used to genotype each SNP on the ABI PRISM 7500 Sequence Detection Systems (Applied Biosystems, Foster City, CA), and emerge the genotypes with Detection Systems software 2.0.1 (Applied Biosystems). The primers and probes for detecting each SNP were self-designed by Primer Express 3.0 (Applied Biosystems) and synthesized by Shanghai GeneCore Biotechnologies (Shanghai, China) as listed in **Table S1**. We further randomly selected about 10% samples to perform repeat assay, and the results were 100% concordant. The success rates of genotyping for these polymorphisms were all above 99%.

### PPP2R5E mRNA expression analysis

Because previous study had showed the function of SNP rs11453459->G [36], we focused on testing the biological effect of another SNP rs1255722A >G of PPP2R5E, which has a significant association with lung cancer risk. The mRNA level of *PPP2R5E* was detected in thirty-two lung tumor tissues [32]. Total RNA was extracted using the Trizol Reagent (Invitrogen) and reverse transcribed to complementary DNA using oligo primer and Superscript II (Invitrogen). The mRNA levels of PPP2R5E and an internal reference gene β-actin were measured on the ABI Prism 7500 sequence detection System (Applied Biosystems) using the SYBR- Green method. The primers for *PPP2R5E* were: 5′- TCA GCA CCA ACT ACT CCT CCA -3′ (forward) and 5′- GCC TTG AGA CCT AAA CTG TGA G -3′ (reverse) and for β-actin were: 5′- GGC GGC ACC ACC ATG TAC CCT -3′ and 5′ – AGG GGC CGG ACT CGT CAT ACT -3′. All analyses were performed in a blinded fashion with the laboratory persons unaware of genotyping data and each assay was done in triplicate.

### Statistical analysis

The Hardy-Weinberg equilibrium (HWE) was tested by a goodness-of-fit chi-square test to compare the expected genotype frequencies with observed genotype frequencies in controls. The chi-square test was used to assess the differences in the distribution of the genotypes as well as alleles of each SNP between cases and controls. An unconditional logistic regression model with adjustment for age, sex, smoking status, drinking status and family history of cancer was used to estimate the association between SNPs and cancer risk. The best genetic model of each SNP was chose based on the smallest Akaike's information criterion [37]. The possible interaction between SNPs and surrounding factors on lung cancer risk was assessed by a multiplicative interaction model as when OR 11>OR 10×OR 01, in which OR 11 =  the OR when both factors were present, OR 01 =  the OR when only factor 1 was present, OR 10 =  the OR when only factor 2 was present [38], [39]. The Breslow-Day test was used to test the homogeneity between stratum-ORs. Moreover, the statistical power was calculated by using the PS Software [40]. The One-way ANOVA test and student's *t* test were used to evaluate the differences in *PPP2R5E* expression in tumor tissues among different genotypes. All tests were two-sided by using the SAS software (version 9.3; SAS Institute, Cary, NC). *P*<0.05 was considered statistically significant.

## Results

### Distribution of PP2A subunit genes genotypes and their associations with risk of lung cancer

The genotype frequencies of above SNPs among controls were all in agreement with the Hardy-Weinberg equilibrium (*P*>0.05 for all). As shown in **Table 1**, the logistical regression analysis showed that the -G and GG genotypes of rs11453459->G conferred a 1.29-fold and 1.51-fold increased risks of lung cancer compared to the common – genotype (-G vs. –: odds ratio [OR]  = 1.29, 95% Confidence interval [CI]  = 1.08–1.56, *P* = 0.006; GG vs. –: OR = 1.51, 95% CI = 1.04–2.22, *P* = 0.033), and the AA variant genotype of rs1255722G >A had a 38% increased lung cancer risk (OR = 1.28, 95% CI = 1.07–1.77, *P* = 0.012) in comparison to the common GG genotype, but AG did not. According to the smallest AIC, the effect of rs11453459->G best fitted the dominant model, the rs11453459G variants (−G + GG) exerted a 1.32-fold increased risk of lung cancer (OR = 1.32, 95% CI = 1.11–1.58, *P* = 0.002); while the rs1255722G >A best fitted the recessive genetic model, the rs1255722AA variant had a 27% increased lung cancer risk (OR = 1.27, 95% CI = 1.02–1.57, *P* = 0.031) compared to G genotypes (AG + GG). However, for the other seven SNPs, no significant association between them and lung cancer risk (*P*>0.05 for all). Moreover, the rs11453459G variants were still significantly associated with increased cancer risk after multiple tests (*P*_Bonferroni_  = 0.018), while rs1255722AA was not (*P*_Bonferroni_  = 0.279).

**Table 1:** Associations between the SNPs in candidate PP2A subunits and risk of lung cancer in the discovery set.

SNP	Gene, location	Case a	Control a,b	MAF c		OR *_het_* d (95%CI)	OR *_hom_* d (95%CI)	Trend test *P* value	OR(95%CI) e
				Case	Control				
		n = 1056	n = 1056						
rs13344984T >C	*PPP2R1A*, promoter	849/193/9	846/190/15	0.100	0.105	1.01 (0.81–1.27)	0.63 (0.27–1.44)	0.699	0.96 (0.79–1.17)
rs11453459->G	*PPP2R1A*, promoter	582/392/68	656/342/52	0.253	0.212	**1.29** **(1.08**–**1.56)**	**1.51** **(1.04**–**2.22)**	**0.002**	**1.32** **(1.11**–**1.58)**
rs10421191G >A	*PPP2R1A*, 3′-UTR	658/322/68	639/342/63	0.319	0.224	0.91 (0.76–1.10)	1.03 (0.72–1.47)	0.620	0.93 (0.78–1.11)
rs2850247C >A	*PPP2R1B*, promoter	805/233/10	823/209/10	0.121	0.110	1.14 (0.92–1.41)	1.08 (0.45–2.61)	0.252	1.14 (0.92–1.40)
rs612345A >G	*PPP2R1B*, promoter	628/378/46	599/397/54	0.223	0.241	0.92 (0.77–1.10)	0.82 (0.54–1.23)	0.222	0.92 (0.79–1.06)
rs7840855C >T	*PPP2R2A*, promoter	690/343/12	727/305/20	0.176	0.164	1.18 (0.98–1.42)	0.64 (0.31–1.32)	0.312	1.14 (0.95–1.38)
rs3742424G >C	*PPP2R5C*, coding	560/406/86	555/433/66	0.275	0.268	0.94 (0.78–1.12)	1.30 (0.92–1.83)	0.564	0.99 (0.83–1.17)
rs1255722G >A	*PPP2R5E*, promoter	292/530/231	330/531/191	0.471	0.434	1.13 (0.93–1.38)	**1.38** **(1.07**–**1.77)**	**0.013**	**1.27** **(1.02**–**1.57)**
rs2292283G >A	*PPP2CA*, promoter	349/518/178	353/504/187	0.418	0.421	1.05 (0.88–1.28)	0.97 (0.75–1.25)	0.951	1.03 (0.88–1.24)

Abbreviation: MAF, minor allele frequency; OR *_het_*, heterozygote versus wild-genotype homozygote; OR *_hom_*, variant homozygote versus wild-type homozygote; 3′-UTR, 3′-untranslated region.

a Wild-type homozygote/heterozygote/variant homozygote.

b The observed genotype frequencies among the controls were all in agreement with the Hardy-Weinberg equilibrium in the control subjects (*P*>0.05 for all).

c MAF of the variant allele.

d Data were calculated by unconditional logistic regression, adjusted for age, sex, smoking status, drinking status and family history of cancer.

e OR and 95%CI was calculated based on the bested genetic model.

The associations of the two promising SNPs were further confirmed in the validation set as shown in **Table 2**, the rs11453459G genotypes were significantly associated with an increased risk of lung cancer (OR = 1.28, 95%CI = 1.00–1.63, *P* = 0.048), and the rs1255722AA genotype conferred an increased lung cancer risk compared to other genotypes (OR = 1.32, 95%CI = 0.98–1.77) with a borderline statistically significance (*P* = 0.069). However, the multiple test showed that only the polymorphism rs11453459->G had an approaching significant association with lung cancer risk (*P*_Bonferroni_  = 0.096), while rs1255722G >A had not (*P*_Bonferroni_  = 0.198). We combined the two populations to increase the study power because the homogeneity test showed that the above associations in two sets were homogeneous (*P* = 0.974 for rs11453459->G; *P* = 0.559 for rs1255722G >A). The carriers of rs11453459G genotypes had a 1.31-fold increased lung cancer risk in dominant model (adjusted OR = 1.31; 95% CI = 1.13–1.51; *P* = 2.00×10^−4^; *P*_Bonferroni_  = 4.00×10^−4^), and the carriers of rs1255722AA genotype had a 1.27-fold increased risk of lung cancer in recessive model (OR = 1.27; 95% CI = 1.07–1.51; *P* = 0.007; *P*_Bonferroni_  = 0.014). In addition, the distribution of demographic characteristics and risk factors of the discovery set and validation set were presented in **Table S2**.

**Table 2:** Frequency distribution of the SNP rs11453459->G and rs1255722G >A and their associations with lung cancer risk.

Genotypes/alleles	Validation set			Merged set c		
	Case n (%)	Control a n (%)	Adjusted OR(95% CI) b	Case n (%)	Control a n (%)	Adjusted OR(95% CI) b
Total no. of subjects	503	623		1559	1679	
**rs11453459->G**						
–	288 (57.6)	393 (63.5)	1.00 (ref)	870 (56.4)	1049 (62.9)	1.00 (ref)
-G	183 (36.6)	201 (32.5)	1.25 (0.96–1.61)	575 (37.3)	543 (32.5)	**1.28 (1.10**–**1.48)**
GG	29 (5.8)	25 (4.0)	1.53 (0.87–2.70)	97 (6.3)	77 (4.6)	**1.53 (1.12**–**2.09)**
Trend test *P* value			**0.036**			**1.00×10^−4^**
G allele	0.241	0.203		0.249	0.209	
Dominant model						
–	288 (57.6)	393 (63.5)	1.00 (ref)	870 (56.4)	1049 (62.9)	1.00 (ref)
G (-G + GG)	212 (42.4)	226 (36.5)	**1.28 (1.00**–**1.63)**	672 (43.6)	620 (37.1)	**1.31 (1.13**–**1.51)**
**rs1255722G >A**						
GG	146 (29.1)	182 (29.2)	1.00 (ref)	438 (28.2)	512 (30.6)	1.00 (ref)
AG	245 (48.9)	325 (52.2)	0.95 (0.72–1.25)	775 (49.9)	856 (51.1)	1.06 (0.91–1.25)
AA	110 (22.0)	116 (18.6)	1.25 (0.88–1.77)	341 (21.9)	307 (18.3)	**1.32 (1.08**–**1.61)**
Trend test *P* value			0.276			**0.010**
A allele	0.464	0.447		0.469	0.439	
Recessive model						
AG + GG	391 (78.0)	507 (81.4)	1.00 (ref)	1213 (78.1)	1368 (81.7)	1.00 (ref)
AA	110 (22.0)	116 (18.6)	1.32 (0.98–1.77)	341 (21.9)	307 (18.3)	**1.27 (1.07**–**1.51)**

a The observed genotype frequencies among the controls were all in agreement with the Hardy-Weinberg equilibrium in both sets (*P*>0.05 for all).

b Adjusted in a logistic regression model that included age, sex, smoking status, drinking status, and family history of cancer.

c The merged set comprised the discovery set and the validate set.

### Combined genotypes and lung cancer risk

As shown in **Table 3**, we combined the risk genotypes of the two SNPs based on the number of risk genotypes (i.e., rs11453459G and rs1255722AA genotypes). We defined that the carriers with rs11453459– and rs1255722G genotypes have zero risk genotype; the carriers with rs11453459– and rs1255722AA, or rs11453459G and rs1255722G genotypes have one risk genotype; and the carriers with rs11453459G and rs1255722AA genotypes have two risk genotypes. We found that compared with the zero risk genotypes carriers, the one and two number of risk genotypes were associated with increased risks of lung cancer in a dose-dependent manner (OR = 1.32, 95% CI = 1.14–1.53 for one, OR = 1.59, 95%CI = 1.23–2.06 for two risk genotypes; *P*_trend_  = 5.63×10^−6^).

**Table 3:** Stratification analysis of the association between number of risk genotypes and lung cancer risk by selected variables.

Variables	Cases			Controls			Adjusted OR (95% CI) a				
	0 n (%)	1 n (%)	2 n (%)	0 n (%)	1 n (%)	2 n (%)	0	1	2	*P*_trend_ b	*P*_inter_ c
Totally	683 (44.3)	702 (45.5)	157 (10.2)	864 (51.8)	681 (40.8)	124 (7.4)	1.00 (ref.)	1.32 (1.14–1.53)	1.59 (1.23–2.06)	**5.63×10^−6^**	
Age (years)											
≤60	360 (45.1)	357 (44.7)	82 (10.3)	455 (52.1)	351 (40.2)	67 (7.7)	1.00 (ref.)	1.30 (1.06–1.59)	1.52 (1.07–2.17)	**0.003**	0.834
>60	323 (43.5)	345 (46.4)	75 (10.1)	409 (51.4)	330 (41.5)	57 (7.2)	1.00 (ref.)	1.33 (1.07–1.64)	1.66 (1.14–2.42)	**0.001**	
Sex											
Male	489 (45.4)	482 (44.7)	107 (9.9)	622 (52.8)	476 (40.4)	80 (6.8)	1.00 (ref.)	1.32 (1.11–1.57)	1.70 (1.24–2.33)	**5.62×10^−5^**	0.633
Female	194 (41.8)	220 (47.4)	50 (10.8)	242 (49.3)	205 (41.8)	44 (9.0)	1.00 (ref.)	1.33 (1.02–1.74)	1.40 (0.89–2.20)	**0.034**	
Family history of cancer											
Yes	52 (40.3)	64 (49.6)	13 (10.1)	79 (55.6)	55 (38.7)	8 (5.6)	1.00 (ref.)	1.82 (1.08–3.05)	3.06 (1.13–8.26)	**0.005**	0.157
No	631 (44.7)	638 (45.2)	144 (10.2)	785 (51.4)	626 (41.0)	116 (7.6)	1.00 (ref.)	1.29 (1.10–1.50)	1.53 (1.17–2.00)	**7.91×10^−5^**	
Family history of lung cancer											
Yes	20 (38.5)	26 (50.0)	6 (11.5)	22 (51.2)	19 (44.2)	2 (4.7)	1.00 (ref.)	1.34 (0.53–3.40)	4.17 (0.66–26.59)	0.158	0.422
No	663 (44.5)	676 (45.4)	151 (10.1)	842 (51.8)	662 (40.7)	122 (7.5)	1.00 (ref.)	1.32 (1.13–1.53)	1.56 (1.20–2.02)	**1.56×10^−5^**	
Smoking status											
Yes	372 (45.9)	355 (43.8)	84 (10.4)	418 (55.1)	288 (37.9)	53 (7.0)	1.00 (ref.)	1.37 (1.11–1.69)	1.78 (1.23–2.58)	**2.00×10^−4^**	0.418
No	311 (42.5)	347 (47.5)	73 (10.0)	446 (49.0)	393 (43.2)	71 (7.8)	1.00 (ref.)	1.28 (1.04–1.57)	1.44 (1.00–2.07)	**0.008**	
Pack years smoked											
0	311 (42.5)	347 (47.5)	73 (10.0)	446 (49.0)	393 (43.2)	71 (7.8)	1.00 (ref.)	1.28 (1.04–1.57)	1.44 (1.00–2.07)	**0.008**	0.514
<20	96 (48.2)	86 (43.2)	17 (8.5)	160 (56.5)	106 (37.5)	17 (6.0)	1.00 (ref.)	1.35 (0.92–1.99)	1.62 (0.79–3.34)	0.07	
≥20	276 (45.1)	269 (44.0)	67 (10.9)	258 (54.2)	182 (38.2)	36 (7.6)	1.00 (ref.)	1.37 (1.06–1.77)	1.69 (1.09–2.64)	**0.003**	
Drinking status											
Yes	124 (42.8)	134 (46.2)	32 (11.0)	191 (56.8)	127 (37.8)	18 (5.4)	1.00 (ref.)	1.76 (1.25–2.49)	2.72 (1.44–5.16)	**7.67×10^−5^**	**0.034**
No	559 (44.6)	568 (45.4)	125 (10.0)	673 (50.5)	554 (41.6)	106 (8.0)	1.00 (ref.)	1.25 (1.06–1.47)	1.42 (1.07–1.88)	**0.002**	
Histological types											
Adenocarcinoma	260 (42.7)	290 (47.6)	59 (9.7)	864 (51.8)	681 (40.8)	124 (7.4)	1.00 (ref.)	1.42 (1.16–1.72)	1.54 (1.09–2.16)	**3.00×10^−4^**	
Squamous cell carcinoma	250 (48.0)	217 (41.7)	54 (10.4)				1.00 (ref.)	1.13 (0.92–1.40)	1.49 (1.05–2.12)	**0.029**	
Large cell carcinoma	27 (41.5)	32 (49.2)	6 (9.2)				1.00 (ref.)	1.57 (0.93–2.65)	1.61 (0.65–4.00)	0.100	
Small cell lung cancer	83 (43.7)	86 (45.3)	21 (11.1)				1.00 (ref.)	1.34 (0.98–1.85)	1.78 (1.06–2.99)	**0.013**	
Other carcinomas	56 (39.2)	70 (49.0)	17 (11.9)				1.00 (ref.)	1.60 (1.11–2.31)	2.12 (1.19–3.76)	**0.002**	
Stages											
I	81 (40.9)	100 (50.5)	17 (8.6)	864 (51.8)	681 (40.8)	124 (7.4)	1.00 (ref.)	1.60 (1.17–2.19)	1.47 (0.84–2.56)	**0.009**	
II	68 (46.9)	65 (44.8)	12 (8.3)				1.00 (ref.)	1.24 (0.86–1.77)	1.23 (0.65–2.35)	0.261	
III	233 (46.9)	220 (44.3)	44 (8.9)				1.00 (ref.)	1.28 (1.04–1.59)	1.38 (0.95–2.01)	**0.014**	
IV	311 (43.7)	317 (44.5)	84 (11.8)				1.00 (ref.)	1.30 (1.08–1.57)	1.85 (1.36–2.51)	**2.44×10^−5^**	

a Compared with 0 risk genotype, ORs were adjusted for age, sex, smoking status, alcohol use, and family history of cancer in a logistic regression models.

b Trend test for lung cancer risk, with number of risk genotypes in each stratum.

c Interaction test P value.

### Stratification analysis of the number of risk genotypes and lung cancer risk

We performed stratification analysis to evaluate the effect of surrounding factors on associations between increased number of risk genotypes and lung cancer risk. As shown in **Table 3**, the associations were significant in all subgroups except for in individuals with a family history of lung cancer, smokers who smoked less than 20 pack-years, subjects whose histological types is large cell carcinoma and in stage II, which may be due to the limitation of small sample sizes in these subgroups. Furthermore, we observed a positively significant interaction between number of *PPP2R1A* and *PPP2R5E* risk genotypes and drinking status on increasing lung cancer risk (*P* = 0.034, **Table S3**).

### Association between the rs1255722G>A genotypes and mRNA levels of PPP2R5E gene

**Figure 1:**
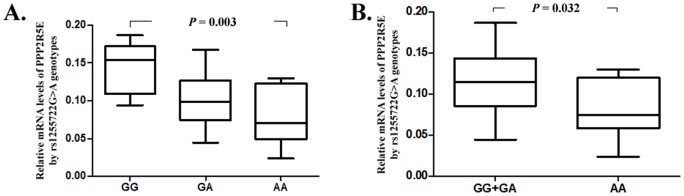
Association between the rs1255722G >A genotypes and relative mRNA levels of PPP2R5E.

As shown in **Figure 1**, the mRNA levels of PPP2R5E in tissues with rs1255722AA genotype were significantly lower than those with G genotypes (ANOVA test: *P* = 0.003). The dichotomized analysis showed that the AA genotype was significantly associated with a decreased mRNA level of PPP2R5E compared to G genotypes (Student's *t* test: *P* = 0.032).

### Bioinformatics Analysis

We further performed bioinformatics analysis to predict the biological effect of rs1255722G >A on affecting the binding ability of potent transcriptional factors by using TFSEARCH (http://www.cbrc.jp/research/db/TFSEARCH.html). The software showed that the G to A transversion may result in a loss binding of a transcription factor c-Ets.

## Discussion

In current two-stage case-control studies of 1,559 lung cancer cases and 1,679 controls conducted in southern and eastern Chinese populations, we found that the rs11453459G genotypes of *PPP2R1A* and rs1255722AA genotype of *PPP2R5E,* and their combined genotypes conferred increased risks of lung cancer. Both the two SNPs were functional as that the rs1255722AA genotype exerted a significantly decreased expression of *PPP2R5E* in lung tumor tissues in comparison to G genotypes, and rs11453459G genotype decreased *PPP2R1A* expression as previously described [36]. For the other SNPs of PP2A subunit genes, we did not observe any significant associations between them and lung cancer risk. To the best of our knowledge, this is the first report on the genetic variants in PP2A subunit genes and lung cancer susceptibility.

The structure A subunit PPP2R1A and regulatory B subunit PPP2R5E are commonly expressed in lung [24], [28]_ENREF_36. They can dephosphorylate several oncogenic kinases via formation of PP2A complex [9]. The frequently genetic mutations and loss-of-function of them in tumors (e.g., lung carcinoma) suggested them to be tumor suppressors [41]–[43]. One previous study has identified that the SNP rs11453459->G can result in low transcriptional activity and decrease *PPP2R1A* expression in lung tissues [36]. Here, we consistently found that the SNP rs1255722G >A could significantly decreased *PPP2R5E* expression in lung tumor tissues, because the G to A transversion may cause a loss binding of a transcription factor c-Ets as the bioinformatics analysis shown. Interestingly, it is reported that c-Ets acts as transcription enhancers promoting PP2A expression in human [44]. Therefore, it is biologically conceivable that the two SNPs were associated with increased risk and their combination cause a much higher risk of lung cancer, because they may cause dysfunctional PP2A.

Moreover, we observed a positively significant interaction between the number of risk genotypes and drinking on increasing lung cancer risk. It is well known that long time alcohol consumption is a potent cancer risk factor [45], and ethanol drinking is a stimulus of PP2A activity [46], the SNP-induced low *PPP2A* expression may cause more adverse effect in response to ethanol stimulation and thus interacted with drinking on lung carcinogenesis.

Genetic variants in *PPP2R1A* or *PPP2R5E* had been reported to be associated with risk of human cancers. Several SNPs in *PPP2R1A* were reported to associated with various cancer risk including breast cancer [22] and uterine serous carcinoma [41]. Similarly, *PPP2R5E* SNPs are susceptible loci for risk of breast cancer [47], lymphocytic leukemia [48], and soft tissue sarcoma [49]. However, these SNPs are all located in introns. Our study was unique and revealed two functional SNPs in *PPP2R1A* and *PPP2R5E* were associated with increased risk of lung cancer. Anyway, all these implicated the SNPs in *PPP2R1A* and *PPP2R5E* are involved in tumorgenesis, suggesting that the variants in *PPP2R1A* and *PPP2R5E* may be valuable biomarkers to predict risk of cancer.

Because this study is a hospital-based case-control study restricted on Chinese Han populations, some limitations are unavoidable (e.g., selection bias). However, the genotype frequencies among controls fitted the Hardy-Weinberg disequilibrium law suggested the randomness of subject selection. And the study powers were acceptable, we have achieved a 95.5% study power (two-sided test, α = 0.05) to detect an OR of 1.31 for the rs11453459G genotypes (37.1% in the controls), and 88.3% study power to detect an OR of 1.27 for the rs1255722AA genotype (which occurred at a frequency of 18.3% in the controls). Meanwhile, the associations were also functional possible. Moreover, results from the GWAS also showed that the frequency distribution of SNP rs1255722G >A was significantly different between cases and controls (rs1255722: *P* = 0.014) [23], but the SNP rs11453459->G was not included in the Affymetrix® Genome-Wide Human SNP Array 6.0. Thus, it appears that our finding that the associations between PP2A subunit gene variants and increased risk of lung cancer is unlikely to have been achieved by chance.

In conclusion, our data suggested that the two functional SNPs (rs11453459->G of *PPP2R1A* and rs1255722A >G of *PPP2R5E*) are associated with risk of lung cancer in Chinese. The identification and description of these two SNPs may lead to their use as genetic biomarker like personalized prevention and therapeutic strategy. Validations with larger population-based studies in different ethnic groups are warranted.

## Supporting Information

Table S1 **Primary information on the TAQMAN assay of nine SNPs in PP2A subunit genes.**
(DOC)

Table S2 **Frequency distributions of selected variables in lung cancer patients and cancer-free controls.**
(DOC)

Table S3 **The interaction between the number of risk genotypes and drinking on increasing lung cancer risk by a multiple interaction analysis.**
(DOC)
