# Supplementary material for: Functional Genetic Polymorphisms in PP2A Subunit Genes Confer Increased Risks of Lung Cancer in Southern and Eastern Chinese
Source: PLoS One. 2013 Oct 29;8(10):e77285. doi: 10.1371/journal.pone.0077285 (PMC3812212; doi:10.1371/journal.pone.0077285)
Supplement: Table S1 — Primary information on the TAQMAN assay of nine SNPs in PP2A subunit genes. (DOC) [file pone.0077285.s001.doc]

**Table S1.** Primary information on the TAQMAN assay of nine SNPs in PP2A subunit genes.

| rs no. | Primers | Probes *^a^* | Color(genotype) |
| --- | --- | --- | --- |
| rs10421191G>A | GGTATCTATTCTCCCCTTCCTAATGTAG(forward) | HEX- AGCTCTGATTTTTA*G*CTG -MGB | Blue (GG) |
|  | CCACTTCTGTTCAACATCTTTTTGG(reverse) | FAM- TCTGATTTTTA*A*CTGGGC -MGB | Red (AG) |
|  |  |  | Green(AA) |
| rs13344984T>C | AGCTTAGCCCTGGGTAGCG (forward) | HEX- TTCCTTCT*T*CTCCCAGCA -MGB | Blue (TT) |
|  | CCTCATTGCGGAGTTCGTC (reverse) | FAM- TTCCTTCT*C*CTCCCAGCA -MGB | Red (CT) |
|  |  |  | Green(CC) |
| rs11453459->G | TGGCATAAATCAAGCACAAA (forward) | HEX-AAGCCTTCCC*-*GGGGA-MGB | Blue (--) |
|  | ACGACCGAGACGAGCAGT (reverse) | FAM- CCTTCCC*G*GGGGA-MGB | Red (-/G) |
|  |  |  | Green(GG) |
| rs2850247 C>A | CACGCTGGTTTAGCCGAAGT | HEX-TGGGGAGCCG*C*GTT-MGB | Blue (CC) |
|  | ATGAATCCCTGTCCCTGTTT | FAM- TGGGGAGCCG*A*GTT-MGB | Red (AC) |
|  |  |  | Green(AA) |
| rs612345 A>G | CGACGGATGACAGTTTTA | HEX- TTAGCTGT*A*TAAGAACTAG -MGB | Blue (AA) |
|  | ACGCGTTACCATGTTTAG | FAM- TAGCTGT*G*TAAGAACTAGA -MGB | Red (AG) |
|  |  |  | Green(GG) |
| rs7840855C>T | AGACATAGGACCCCATGAATCCT(forward) | HEX- CACGTAAACTGGA*C*TC -MGB | Blue (CC) |
|  | AGGCCGCGGCTGTGT (reverse) | FAM- CACGTAAACTGGA*T*TC -MGB | Red (CT) |
|  |  |  | Green(TT) |
| rs3742424G>C | CTTAGTTTGTAGCCAACGC(forward) | HEX- CACTGCAGG*G*CCGA -MGB | Blue (GG) |
|  | GATTGGTATGGCACAGGA(reverse) | FAM- CACTGCAGG*C*CCGA -MGB | Red (GC) |
|  |  |  | Green(CC) |
| rs1255722A>G | TGGGTCTTTCCGAGCTCTGA(forward) | HEX- CAGTCGGCTGAC*A*GG-MGB | Blue (AA) |
|  | CCACAGTTTGCAGTCTATTCATTCA(reverse) | FAM- AGTCGGCTGAC*G*GG-MGB | Red (AG) |
|  |  |  | Green(GG) |
| rs2292283A>G | CCCAGTCTGGTAAATTAAGTATGTGATAA(forward) | HEX-TTCTAGAAGAGTAGAT*A*TAAGGT -MGB | Blue (AA) |
|  | CCTCCCATTAGTGTGCACAAAA (reverse) | FAM- TTCTAGAAGAGTAGAT*G*TAAG-MGB | Red (AG) |
|  |  |  | Green(GG) |

*^a^* Red italic nucleotides indicate the polymorphic sites in probes.
