# Supplementary material for: Functional Genetic Polymorphisms in PP2A Subunit Genes Confer Increased Risks of Lung Cancer in Southern and Eastern Chinese
Source: PLoS One. 2013 Oct 29;8(10):e77285. doi: 10.1371/journal.pone.0077285 (PMC3812212; doi:10.1371/journal.pone.0077285)
Supplement: Table S2 — Frequency distributions of selected variables in lung cancer patients and cancer-free controls. (DOC) [file pone.0077285.s002.doc]

**Table S2.**Frequency distributions of selected variables in lung cancer patients and cancer-free controls.

| Variables |  | Discovery set (Southern Chinese) | | |  | Validation set (Eastern Chinese) | | |  | Merged set | | |
| --- | --- | --- | --- | --- | --- | --- | --- | --- | --- | --- | --- | --- |
|  |  | Case  (n=1056)  n(%) | Control  (n=1056)  n(%) | *P* ***^a^*** |  | Case  (n=503)  n(%) | Control  (n=623)  n(%) | *P* ***^a^*** |  | Case  (n=1559)  n(%) | Control  (n=1679)  n(%) | *P* ***^a^*** |
| Age (years) |  |  |  |  |  |  |  |  |  |  |  |  |
| ≤ 60 |  | 536(50.8)  520(49.2) | 534(50.6) | 0.931 |  | 273(54.3) | 343(55.1) | 0.793 |  | 809(51.9) | 877(52.2) | 0.846 |
| > 60 |  |  | 522(49.4) |  |  | 230(45.7) | 280(44.9) |  |  | 750(48.1) | 802(47.8) |  |
| Sex |  |  |  |  |  |  |  |  |  |  |  |  |
| Male |  | 746(70.6) | 746(70.6) | 1.000 |  | 345(66.6) | 433(70.4) | 0.496 |  | 1091(70.0) | 1185(70.5) | 0.710 |
| Female |  | 310(29.4) | 310(29.4) |  |  | 158(31.4) | 184(29.6) |  |  | 468(30.0) | 494(29.5) |  |
| Family history of cancer |  |  |  |  |  |  |  |  |  |  |  |  |
| Yes |  | 104(9.9) | 103(9.8) | 0.942 |  | 25(5.0) | 44(7.1) | 0.046 |  | 129(8.3) | 147(8.8) | 0.625 |
| No |  | 952(90.1) | 953(90.2) |  |  | 478(95.0) | 579(92.9) |  |  | 1430(91.7) | 1532(91.2) |  |
| Family history of lung cancer |  |  |  |  |  |  |  |  |  |  |  |  |
| Yes |  | 42(4.0) | 30(2.8) | 0.150 |  | 10(2.0) | 13(2.1) | 0.907 |  | 52(3.3) | 43(2.6) | 0.192 |
| No |  | 1014(96.0) | 1026(97.2) |  |  | 493(98.0) | 610(97.9) |  |  | 1507(96.7) | 1636(97.4) |  |
| Smoking status |  |  |  |  |  |  |  |  |  |  |  |  |
| Current |  | 394(37.3) | 366(34.6) | 0.028 |  | 118(23.5) | 168(26.6) | 1.79×10^-7^ |  | 512(32.8) | 532(31.7) | 1.48×10^-6^ |
| Former |  | 207(19.3) | 176(16.8) |  |  | 105(20.9) | 57(9.2) |  |  | 312(20.0) | 233(13.9) |  |
| Never |  | 455(43.1) | 514(48.7) |  |  | 280(55.6) | 400(64.2) |  |  | 735(47.2 | 914(54.4) |  |
| Pack-years smoked |  |  |  |  |  |  |  |  |  |  |  |  |
| ≥20 |  | 459(43.5) | 314(29.7) | 9.40×10^-12^ |  | 165(32.8) | 165(26.5) | 0.011 |  | 624(40.0) | 479(28.5) | 1.94×10^-11^ |
| <20 |  | 142(13.4) | 228(21.6) |  |  | 58(11.5) | 58(9.3) |  |  | 200(12.8) | 286(17.0) |  |
| 0 |  | 455(43.1) | 514(48.7) |  |  | 280(55.7) | 400(64.2) |  |  | 735(47.2) | 914(54.5) |  |
| Sex and smoking subgroups |  |  |  |  |  |  |  |  |  |  |  |  |
| Male smokers |  | 583(55.2) | 510(48.3) | 5.14×10^-5^ |  | 176(35.0) | 215(34.5) | 4.77×10^-9^ |  | 759(48.7) | 725(43.2) | 9.35×10^-6^ |
| Male non-smokers |  | 163(15.4) | 236(22.6) |  |  | 169(33.6) | 224(36.0) |  |  | 332(21.3) | 460(27.4) |  |
| Female smokers |  | 18(1.7) | 32(3.0) |  |  | 47(9.3) | 8(1.3) |  |  | 65(4.2) | 40(2.4) |  |
| Female non-smokers |  | 292(27.7) | 278(26.3) |  |  | 111(22.1) | 176(28.2) |  |  | 403(25.8) | 454(27.0) |  |
| Drinking status |  |  |  |  |  |  |  |  |  |  |  |  |
| Current |  | 165(15.6) | 186(17.6) | 0.042 |  | 36(7.2) | 75(12.0) | 0.017 |  | 201(13.9) | 261(15.5) | 0.049 |
| Former |  | 64(6.1) | 41(3.9) |  |  | 28(5.6) | 40(6.4) |  |  | 92(5.9) | 81(4.8) |  |
| Never |  | 827(78.3) | 829(78.5) |  |  | 439(87.2) | 508(81.6) |  |  | 1266(81.2) | 1337(79.7) |  |
| Sex and drinking subgroups |  |  |  |  |  |  |  |  |  |  |  |  |
| Male drinkers |  | 214(20.3) | 201(19.0) | 0.293 |  | 51(10.1) | 107(17.2) | 0.003 |  | 265(17.0) | 308(18.3) | 0.721 |
| Male non-drinkers |  | 532(50.4) | 545(51.6) |  |  | 294(58.5) | 332(53.3) |  |  | 826(53.0) | 877(52.2) |  |
| Female drinkers |  | 15(1.4) | 26(2.5) |  |  | 13(2.6) | 8(1.3) |  |  | 28(1.8) | 34(2.0) |  |
| Female non-drinkers |  | 295(27.9) | 284(26.9) |  |  | 145(28.8) | 176(28.2) |  |  | 440(28.2) | 460(27.4) |  |
| Histological types |  |  |  |  |  |  |  |  |  |  |  |  |
| Adenocarcinoma |  | 384(36.4) |  |  |  | 231(45.9) |  |  |  | 615(39.4) |  |  |
| Squamous cell carcinoma |  | 369(34.9) |  |  |  | 158(31.4) |  |  |  | 527(33.8) |  |  |
| Large cell carcinoma |  | 43(4.1) |  |  |  | 23(4.6) |  |  |  | 66(4.2) |  |  |
| Small cell lung cancer |  | 128(12.1) |  |  |  | 65(12.9) |  |  |  | 193(12.4) |  |  |
| Other carcinomas *^b^* |  | 132(12.5) |  |  |  | 26(5.2) |  |  |  | 158(10.2 ) |  |  |
| Stages |  |  |  |  |  |  |  |  |  |  |  |  |
| I |  | 154(14.6) |  |  |  | 46(9.2) |  |  |  | 200(12.8) |  |  |
| II |  | 94(8.9) |  |  |  | 53(10.5) |  |  |  | 147(9.5) |  |  |
| III |  | 333(31.5) |  |  |  | 157(31.2) |  |  |  | 490(31.4) |  |  |
| IV |  | 475(45.0) |  |  |  | 247(49.1) |  |  |  | 722(46.3) |  |  |

***^a^*** *P* values for a χ^2^ test.

***^b^*** Mixed-cell or undifferentiated carcinoma.
