# Supplementary material for: Functional Genetic Polymorphisms in PP2A Subunit Genes Confer Increased Risks of Lung Cancer in Southern and Eastern Chinese
Source: PLoS One. 2013 Oct 29;8(10):e77285. doi: 10.1371/journal.pone.0077285 (PMC3812212; doi:10.1371/journal.pone.0077285)
Supplement: Table S3 — The interaction between the number of risk genotypes and drinking on increasing lung cancer risk by a multiple interaction analysis. (DOC) [file pone.0077285.s003.doc]

**Table S3.** The interaction between the number of risk genotypes and drinking on increasing lung cancer risk by a multiple interaction analysis.

| Variants | OR (95%CI) | *P* value |
| --- | --- | --- |
| Drinking | 0.50(0.31-0.82) | 0.005 |
| Number of risk genotypes | 1.22(1.08-1.37) | 0.002 |
| Drinking **×**Number of risk genotypes | 1.35(1.02-1.79) | 0.034 |
